# Black Tattoos Entail Substantial Uptake of Genotoxicpolycyclic Aromatic Hydrocarbons (PAH) in Human Skin and Regional Lymph Nodes

**DOI:** 10.1371/journal.pone.0092787

**Published:** 2014-03-26

**Authors:** Karin Lehner, Francesco Santarelli, Rudolf Vasold, Randolph Penning, Alexis Sidoroff, Burkhard König, Michael Landthaler, Wolfgang Bäumler

**Affiliations:** 1 Department of Dermatology, University of Regensburg, Regensburg, Germany; 2 Department of Organic Chemistry, University of Regensburg, Regensburg, Germany; 3 Department of Forensic Medicine, Ludwig Maximilian University, Munich, Germany; 4 Department of Dermatology and Venereology, University of Innsbruck, Innsbruck, Austria; University Paris Diderot-Paris 7, France

## Abstract

Hundreds of millions of people worldwide have tattoos, which predominantly contain black inks consisting of soot products like Carbon Black or polycyclic aromatic hydrocarbons (PAH). We recently found up to 200 μg/g of PAH in commercial black inks. After skin tattooing, a substantial part of the ink and PAH should be transported to other anatomical sites like the regional lymph nodes. To allow a first estimation of health risk, we aimed to extract and quantify the amount of PAH in black tattooed skin and the regional lymph nodes of pre-existing tattoos. Firstly, we established an extraction method by using HPLC – DAD technology that enables the quantification of PAH concentrations in human tissue. After that, 16 specimens of human tattooed skin and corresponding regional lymph nodes were included in the study. All skin specimen and lymph nodes appeared deep black. The specimens were digested and tested for 20 different PAH at the same time.PAH were found in twelve of the 16 tattooed skin specimens and in eleven regional lymph nodes. The PAH concentration ranged from 0.1–0.6 μg/cm^2^ in the tattooed skin and 0.1–11.8 μg/g in the lymph nodes. Two major conclusions can be drawn from the present results. Firstly, PAH in black inks stay partially in skin or can be found in the regional lymph nodes. Secondly, the major part of tattooed PAH had disappeared from skin or might be found in other organs than skin and lymph nodes. Thus, beside inhalation and ingestion, tattooing has proven to be an additional, direct and effective route of PAH uptake into the human body.

## Introduction

Polycyclic aromatic hydrocarbons (PAH) such as benzo[a]pyrene (b[a]p) belong to a large class of well-studied chemical pollutants with ubiquitous occurrence in the environment. They consist of two or more fused benzene rings and are generated naturally or notably found as a result of incomplete combustion of organic materials, fossil fuels, vehicular emission or even tobacco smoke. For some time it is well known that human exposure to complex mixtures of PAH occurs primarily through three routes: (i) the respiratory tract through the smoking of tobacco products and the inhalation of polluted air, (ii) the gastrointestinal tract through the ingestion of contaminated drinking water and food, and (iii) skin contact, which usually occurs from occupational exposure [1].OnePAH isclassified by the International Agency of Research in Cancer as human carcinogens (b[a]p) and several others as probably or possibly carcinogenic to humans [2].B[a]p, benz[a]anthracene, benzo[b]fluoranthene,benzo[ghi]perylene, benzo[j]fluoranthene, benzo[k]fluoranthene, chrysene, cyclopenta[cd]pyrene, dibenz[a,h]anthracene,dibenzo[a,e]pyrene, dibenzo[a,h]pyrene, dibenzo[a,i]pyrene,dibenzo[a,l]pyrene, indeno[1,2,3-cd]pyrene and 5-methylchrysenehave shown clear genotoxicity in standard assays in vitro andin vivo [3].

Animal studies and epidemiological studies have associated PAH exposure with multiple adverse health effects invarious organs (e.g. cancer of lung, skin, and bladder, neural tube defects [4]–[10]). This has been frequently linked to mutagenic properties of PAH metabolites. Benzo[a]pyrene has been thoroughly studied and requires usually metabolic activation by cytochrome P450 enzymes through covalent binding to DNA (DNA adduct formation) [11].The active metabolite benzo[a]pyrene-7,8-diol-9,10-epoxide (BPDE) represents probably the ultimate carcinogen [12]. PAH are alsopotent immunotoxic agents that impair functional activation of lymphocytes [13] and inhibit macrophage differentiation [14]. It was shown in experimental animal and human studies that diesel exhaust particles (DEP) enhance allergic antibody (IgE) production via PAH induced mechanisms, in particular by phenanthrene [15]. Due to the production process of black tattoo inks, it is not surprising that both, DEP and black tattoo inks contain comparable PAH species.

In our previous studies, a new source of PAH intake for humans was discovered by chemical analysis of commercially available black tattoo inks. 20 different PAH and phenol could be quantitatively detected in black tattoo suspensions using an established extraction procedure with HPLC – DAD technique and the method of internal standard. The amount of extracted PAH was in the range of 0.14 to 201.00 μg/g [16]. This is an alarm signal since millions of people have many and large tattoos, which are predominantly black [17]. Regulation of ink composition is frequently missing. Black tattoo inks mainly consist of Carbon Black, a mixture of different solvents and other ingredients, whereas the actual composition may vary for the different ink products. Carbon Black itself is already listed by IARC as possibly carcinogenic to humans (group 2 B) [18]. In addition, the black inks, which are placed in the skin, are partially transported in the human body via lymphatic system and can be also found in the regional lymph nodes [19].

Thus, the present study was designed to analyse the amount of PAH and phenol in real black tattooed human skin as well as in the corresponding regional lymph nodes by using our established extraction procedure and HPLC - DAD technology.

## Materials and Methods

### Chemicals and reagents

20 well known PAH (purity ∼99%) were obtained from Sigma Aldrich (Steinheim, Germany): naphthalene, acenaphthylene, acenaphthene, fluorene, phenanthrene, anthracene, fluoranthene, pyrene, benzo[a]anthracene, chrysene, benzo[b]fluoranthene, benzo[k]fluoranthene, benzo[a]pyrene, dibenzo[a,h]anthracene, benz[ghi]perylene, indeno[1,2,3-cd]pyrene, dibenzo[a,e]pyrene, dibenzo[a,l]pyrene, 5-methylchrysene and benzo[j]fluoranthene. Phenol (purity >99%) as analytical reference was obtained from Riedel-de Haen. For the internal standard (ISTD), 9,10-diphenylanthracene (purity >99%) was obtained from Riedel-de Haen. ATL buffer and proteinase K (>600 mAU/mL) were purchased from Qiagen (Hilden, Germany).

One milligram of each 20 PAH and phenol was dissolved in one milliliter of acetonitrile to obtain a 1.0 mg/mL PAH stock solution and treated by ultrasound (BandelinSonorex Super RK 103 H) for 10 min, respectively. 200 μL of each compound stock solution were combined and filled up with acetonitrile to obtain a concentration of 0.01 mg/mL. As internal standard (ISTD), 9,10-diphenylanthracene (9,10 DPA) was prepared as a 0.08 mg / mL stock solution in acetonitrile. The solvents benzene, acetonitrile, and acetone were of liquid chromatography quality (LiChroSolv, Merck, Darmstadt, Germany). Millipore water as solvent A for HPLC analysis was freshly produced by a Milli-Q Advantage A10 system (TOC 5 ppb, Millipore, Molsheim, Ce'dex).

### Skin preparation and related regional lymph nodes

For recovery experiments, human skin was anonymously obtained from surgical excisions (Department of Dermatology, University of Regensburg, Germany). 16samples of tattooed skin and regional lymph nodes were taken from dead persons at the Department of Forensic Medicine at the Ludwig Maximilians University Munich along with court-ordered autopsies. No cosmetic impairment occurred during the skin harvest because the samples were taken from the periphery of large injuries of heavily traumatized bodies, for instance from people killed by train accidents. All samples were anonymously obtained.

Skin sampling was performed as soon as possible after exitus and frozen to −80°C. The skin was chopped up to slices at a size of 1 cm^2^ and placed into Eppendorf cups (Eppendorf, Wesseling-Berzdorf, Germany), and 400 μL of PBS (PAA, Pasching, Austria) was added. Proteins were denatured by heating at 95°C for 5 min. After cooling to room temperature, a total of 180 μL of buffer ATL and 20 μL of proteinase K were added to the skin, mixed by vortex and incubated at 55 °C until the tissue was completely lysed. The respective regional lymph nodes were weighted, cleaved and treated in an analogue work-up procedure. The experiments were performed according to the Helsinki Declaration of 1975. The study was approved by the local ethic committee at University Hospital of Regensburg in September 2007. In this approval, the local ethic committee waived the need for consent of sample donors.

Skin and lymph node biopsies were taken from specimens for histological examination using normal hematoxylin andeosin staining (H&E) and a standard histology microscope.

### Extraction procedure

For recovery studies, one milliliter of the stock solution containing the respective 21 reference substances was added to 0.6 mL of digested human skin in a glass test tube (8 mm 10 mm, NS 14; Neubert-Glas, Ilmenau, Germany). For tattooed tissue, 0.6 mL of digested tattooed human skin and 0.6 mL of digested regional lymph node were placed in a glass test tube (8–10 mm, NS 14; Neubert-Glas, Ilmenau, Germany), respectively. The extraction solvents benzene/acetone (2 mL/1 mL) were added. The extraction of PAH and phenol from the tattooed digested human skin was done using an alternating combination of vortex mixer (MS 1 Minishaker, IKA, Brazil) and ultrasonic bath (BandelinSonorex Super RK 103 H) for 1/5/1/5/1 minutes. After centrifugation at 4°C with 2500 ***g*** (Eppendorf Centrifuge, 5702 RH), the supernatant was collected, 100 μL of keeper “Diglyme” was added to avoid the loss of volatile components during the extraction procedure. Finally, the solvent was removed under a gentle stream of nitrogen (2 bar, 20 min, rt) and concentrated to 100 μL. For the HPLC analysis, each sample, consisting of the extracted compounds concentrated in keeper, was reconstituted in 0.3 mL of acetonitrile, filtered through a PTFE-filter (CHROMAFIL, O-20/15, organic, pore size 0.2 μm, Machery-Nagel, Düren, Germany) and finally 50 μL of internal standard stock solution were added. After the final step, the total volume of each sample was 0.45 mL.The recovery experiments were done in triplicate and the values are presented as mean values ± standard deviation

### HPLC analysis

The calibration solution consisted of 0.3 mL of PAH-phenol stock solution and 50 μL tracer stock solution. All samples were analyzed using a model 1100 HPLC (Agilent Technologies, Waldbronn, Germany) fitted with a C18 analytical column for nanoscalic environmental analysis (PhenomenexEnvironsep PP, particle size 3 μm, 125×2.00 mm, Aschaffenburg, Germany) and diode array detector (DAD). The Injection volume was 10 μL. The data-files were analysed using a HPLC-3D-ChemStation Rev. B.04.02. The PAH and phenol could be separated by gradient elution with water [0.0059 w % trifluoroacetic acid] (solvent A) and acetonitrile (solvent B) at a constant flow rate of 0.3 mL/min. A gradient profile with the following proportions of solvent B was applied [t (min), % B]: (0, 40), (2, 40), (27, 98), (40, 98). The chromatograms were monitored at 220 nm. The concentration of PAH in the solutions was determined by the method of internal standard. For each compound (i), the calibration factor (CF_i_) was determined in a calibration run (single level calibration). The respective concentration of the internal standard was chosen to be in the range of the concentration of the PAH. The values are calculated as follows,
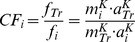
where f_Tr_ is the response-factor of the internal standard (ISTD), m_i_^K^ the mass-concentration of compound i in the solution k, and m_Tr_^K^ the mass-concentration of ISTD in solution k. a_Tr_^K^ is the area of ISTD in solution k and a_i_^K^ the area of compound i in solution k.

## Results

### PAH recovery from human tissue – recovery experiments

The goal of the recovery experiments was the establishment of an optimal procedure to extract a selection of 20 important PAH and phenol from human tissue. The used substances are listed by the United States Environmental Protection Agency (US EPA) due to their toxicity and carcinogenicity (USEPA, 1982; National Toxicology Program, 1998; Warshawsky, 1999) or Scientific Committee on Food (SCF) of health and consumer protection directorate-general in Europe.

**Figure 1:**
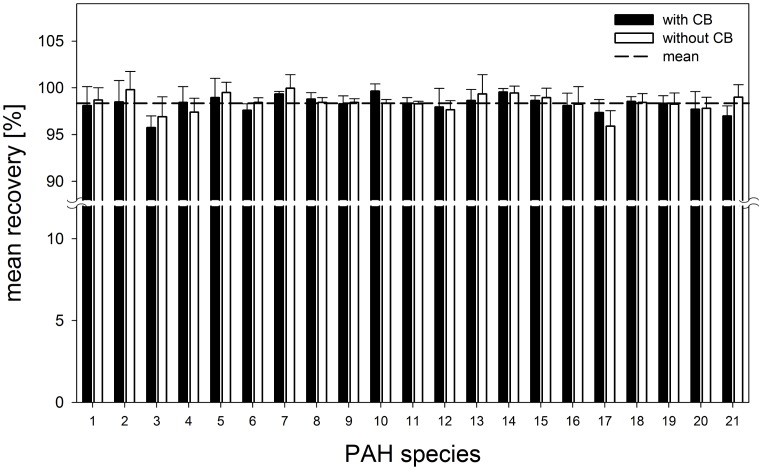
Recovery of phenol (1) and PAH (2–21) from digested human skin using a combination of vortex and ultrasonic extraction in a 2∶1 benzene/acetone solution and keeper without (white) and loaded with (black) Carbon Black particles. Recovery ranges from 96–99% for both, without and with adding Carbon Black particles whereby the mean RSD (n = 3) did not exceed 3%.

The extraction procedure yielded a high recovery rate for each single substance in the range of 96 to 99%, rather independent of the volatility of the recovered molecule (Figure 1). In a next step, 20 mg of pure Carbon Black was added to the skin suspension together with the PAH/phenol mixture to approach the real skin tattoo conditions. The presence of Carbon Black as sorptive phase for PAH did not affect the recovery rates as shown in Figure 1. The recovery experiment clearly showed that the present procedure is an effective and quantitative method to extract PAH even in the presence of Carbon Black, which are usually present in black inks in original tattoo suspensions.

### PAH in tattooed skin specimen

**Figure 2:**
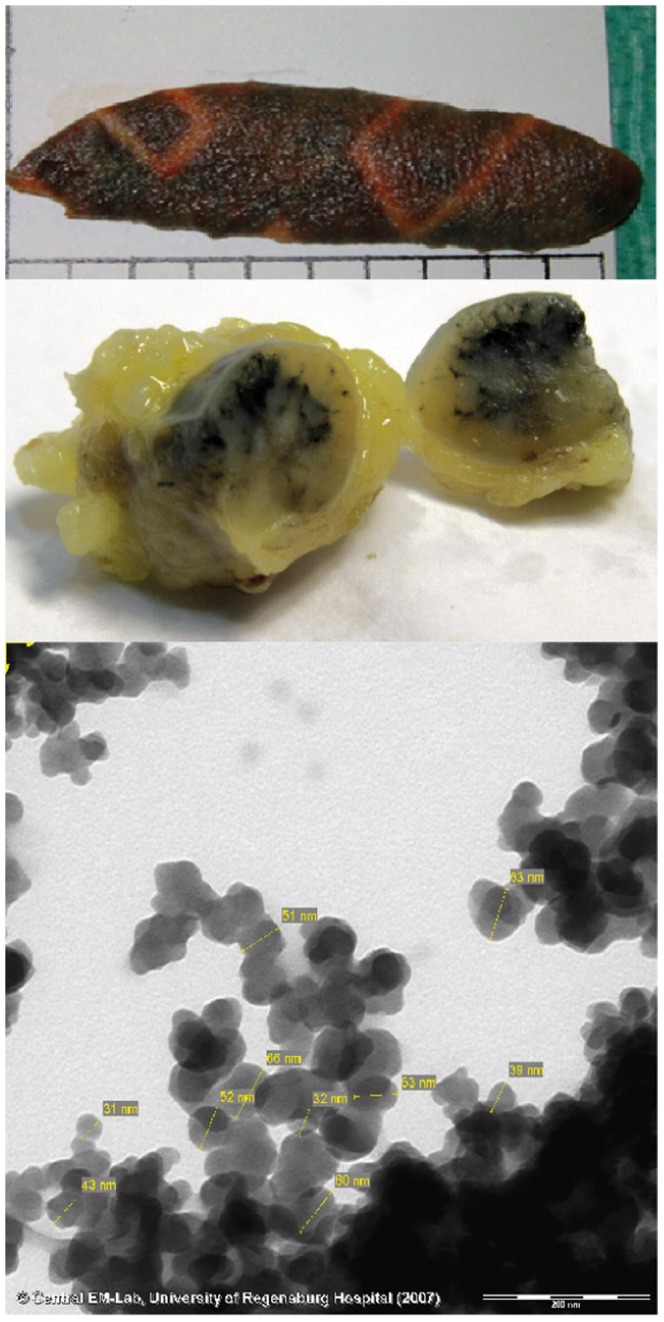
Specimen of black tattooed human skin (top) and one of the corresponding regional lymph node (center), which was cutted into two pieces. Transmission electron microscopy shows the shape and size of black tattoo particles, which consist of Carbon Black nanoparticles (bottom).

**Figure 3:**
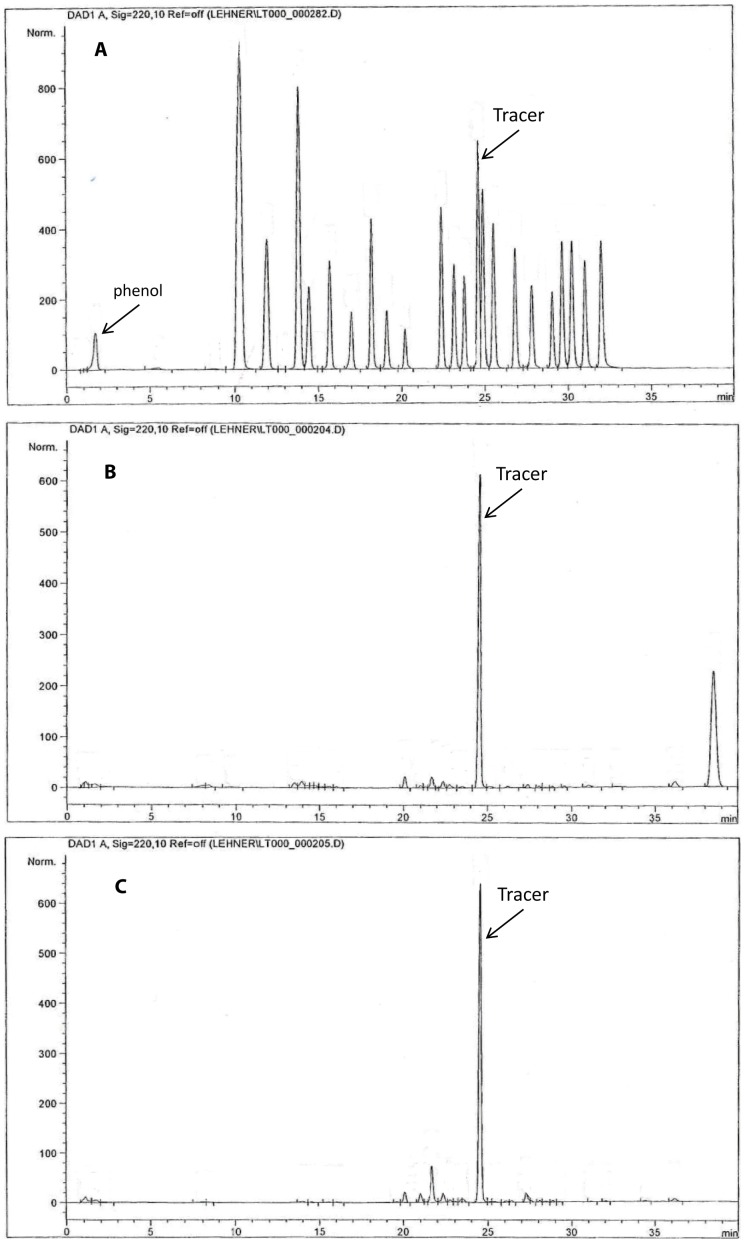
HPLC chromatogramis displayed of all 21 PAH (A), which could be detected at the same time. HPLC chromatogramsare displayed for a skin sample (B), and for a lymph node sample (C).

The concentrations of PAH in 16 samples of tattooed skin were determined, an example of the specimens is shown in Figure 2. These tattoos existed for months or years, the exact time spans could not be evaluated. The size of the tattoo specimens was in the range of 1.8–12.0 cm^2^. Only black tattooed skin areas were used for extraction by excision and the calculated PAH concentration was referred to the size of this area. The extracted and lysed skin samples were screened using HPLC – DAD technology as established in the recovery experiments. Figure 3 exemplarily shows a chromatogram of PAH detected in a skin sample.Using the method of internal standard, the total amount of PAH in twelve of 16 tattooed skin samples ranged from 0.1 to 0.6 μg/cm^2^ (Table 1). As our extraction procedure shows a rather high experimental accuracy, this broad range of values is probably caused by different tattooing procedures using different pigment concentrations together with various admixtures. In fact, differences might also be explained by the tattooed skin site.

**Table 1:** Total Concentration of PAH in skin and lymph node specimens.

Specimen(skin/lymph node)	Total amount of PAH in skin (μg/cm^2^)	Total amount of PAH in lymph node (μg/g)
# 1	0.1	0.1
# 2	0.6	11.8
# 3	-*	-
# 4	0.4	0.9
# 5	0.2	2.3
# 6	0.4	2.9
# 7	0.2	3.1
# 8	0.1	-
# 9	-	-
#10	0.1	0.2
#11	0.1	10.6
#12	0.2	10.2
#13	-	-
#14	-	-
#15	0.2	0.4
#16	0.2	2.2

*below detection limit.

Phenanthrene and acenaphthene were present in seven of 16 skin samples, fluorene, anthracene,benzo[j]fluoranthene and naphthalene were found in two tattoos, respectively. Pyrene, benzo[k]fluoranthene, benzo[b]fluoranthene and fluoranthene could be quantified in almost one specimen. PAH dibenzo[a,e]pyrene, indeno[1,2,3-c,d]pyrene, benzo[g,h,i]perylene, dibenzo[a,h]anthracene, dibenzo[a,l]pyrene, benzo[a]pyrene, 5-methylchrysene, chrysene, benzo[a]anthracene, and acenaphthylene were below the detection limit and could not be detected in any tattoo sample within the experimental accuracy (Table 2). There are some indications for the presence of phenol in few tattoo samples. Phenanthrene was detected with high amounts in both, commercially available black tattoo ink suspensions as previously published and black tattooed human skin (Table 2). There might be some correlation between PAH present in black tattoo inks and PAH punctured into human skin because the same substances found in human matrices were recently detected in tattoo ink suspensions.

**Table 2:** Concentration of single PAH in skin and lymph nodes compared to tattoo inks.

PAH	mean amount ink suspension*[μg/g]	mean amount in skin[μg/cm^2^]	mean amount in lymph nodes[μg/g]	IARC**classification
phenanthrene	24.5	0.1	2.2	3
acenaphthylene	14.5	-***	-	no data
**benzo[b]fluoranthene**	**4.5**	**0.1**	**-**	**2B**
pyrene	4.4	0.1	-	3
anthracene	3.3	0.2	9.7	3
fluoranthene	2.8	0.1	-	3
**chrysene**	**1.7**	**-**	**-**	**2B**
**benzo[a]anthracene**	**1.6**	**-**	**-**	**2B**
**benzo[g,h,i]perylene**	**1.2**	**-**	**-**	**3**
**indeno[1,2,3-cd]pyrene**	**1.1**	**-**	**2.1**	**2B**
acenaphthene	0.9	0.1	0.2	3
fluorene	0.9	0.2	2.5	3
**benzo[k]fluoranthene**	**0.4**	**0.03**	**4.2**	**2B**
**benzo[a]pyrene**	**0.3**	**-**	**-**	**1**
naphthalene	0.3	0.05	0.3	2B
**dibenzo[a,h]anthracene**	**0.1**	**-**	**-**	**2A**
**benzo[j]fluoranthene**	**-**	**0.1**	**2.0**	**2B**
**dibenzo[a,l]pyrene**	**-**	**-**	**-**	**2A**
**5-methylchrysene**	**-**	**-**	**-**	**2B**
**dibenzo[a,e]pyrene**	**-**	**-**	**-**	**3**

*mean PAH values recently detected in commercially available black tattoo ink suspensions [16].

**taken from the fact sheet of the JRC, Polycyclic Aromatic Hydrocarbons.

***below detection limit.

The bold PAH have been proven to be genotoxic in vitro and in vivo according to [3].

### PAH in regional lymph nodes

Due to lymphatic transportation of Carbon Black or unbound PAH, part of PAH molecules may appear also in the regional lymph nodes. Figure 2 shows an example of a sliced lymph node, which contains black tattoo ink. The same PAH extraction and analysis procedures were applied for the lymph nodes now. Figure 3shows a chromatogram of PAH detected in a lymph node. The weight of the 16 lymph nodes specimens ranged from 0.06 to 0.59 g. Quantification of PAH was possible in eleven of the 16 samples (Table 1) and referred to the weight of the lymph node. The concentration of PAH ranged from 0.1–11.8 μg/g. Phenanthrene could be detected in seven samples, acenaphthene in four. benzo[j]fluoranthene was detected two times and anthracene, indeno[1,2,3-c,d]pyrene, fluorene, benzo[k]fluoranthene and naphthalene were quantified in one specimen, each (Table 2). Dibenzo[a,e]pyrene, benzo[g,h,i]perylene, dibenzo[a,h]anthracene, dibenzo[a,l]pyrene, benzo[a]pyrene, benzo[b]fluoranthene, 5-methylchrysene, chrysene, benzo[a]anthracene, pyrene, fluoranthene and acenaphthylene were below the spectral detection limit and could not be sufficiently identified within experimental accuracy according to the applied procedure (Table 2).

The origin of PAH in the lymph nodes and the resulting concentration may not necessarily correlate to the results of skin extraction. For the present study, single lymph node was excised from the adjacency of the respective tattooed skin. It remains unclear which skin area was drained to which extent by which lymph node. Also other regional lymph nodes could contain black tattoo inks and PAH.

## Discussion

The intake of PAH in humans occurs via major pathways such as eating, drinking, and inhalation. Humans inhale PAH along with airborne fine particles, which originate from combustion emissions of vehicles (e.g., diesel exhaust particles, DEP), heating and power sources (e.g., coal, oil) and tobacco smoke [7]. Animal experiments showed that after inhalation of DEP, PAH could be detected in trachea-bronchial lymph nodes even months after exposure to DEP [20]. Smoked meat products show total PAH concentrations in the range of 0.01–19 μg/kg [21]. Based on data from EU surveys, a maximum daily exposure of adults to the most important PAHs may be 5 μg per person [22].

Some decades ago, studies in experimental animals showed that already topical application of coal tar solution caused induction of aryl hydrocarbon hydroxylase in skin and, after percutaneous absorption, in liver as well and that such induced enzymatic activity could relate to carcinogenic responses to this agent [23]. Being produced by imperfect combustion, black tattoo inks likewise contain a complex mixture of various PAH [16]. In contrast to topical coal tar application and a possible penetration into skin, the black inks are injected into skin with solid needles corresponding to an almost complete penetration of PAH into skin.

### Distribution in skin

We recently found up to 200 μg/g of PAH and up to 385 μg/g phenol in commercially available tattoo inks, which mainly consist of Carbon Black nanoparticles [16]. In case of single black tattoo with a typical size of 400 cm^2^, we had estimated an amount of up to 400 μg PAH and 770 μg phenol that is injected in skin. We now have identified another and important pathway of PAH intake to humans, tattooing with black inks.

In addition, a nation-wide survey in Germany revealed that about 28% of tattooed individuals have more than 3 tattoos and 36% have tattoos with 900 cm^2^ in size or even larger [24]. Assuming these percentages also to other nations in US or Europe [17], we have to assume that more than 100 millions of people carry PAH in skin and other organs due to their black tattoos. In addition to PAH, compounds like dibutyl phthalate or dibenzofuran were found in such black tattoo inks [25].

Beside various adverse skin reactions [26], also case reports about skin tumours in tattoos are published and summarized in a recent review article [27]. It is still under controversial discussion, whether these malignancies such as basal cell carcinoma or malignant melanoma coincidentally occurred only.

There are enzymatic and non-enzymatic pathways to form the hazard diol-epoxides of PAH molecules such as benz[a]pyrene-7,8-diol-9,10-epoxide (BPDE) [28]. Both pathways might also occur in tattooed skin. Firstly, cytochrome P450 dependent enzymes can be triggered by injecting foreign material like tattoo inks in skin [29]. Secondly, PAH generate singlet oxygen when tattooed skin is exposed to UV radiation leading to an oxidation of PAH.

Akintobi et al showed induction of cytochrome P450 1B1 (CYP1B1) expression in human dermal fibroblasts when exposed to xenobiotic substances like 2,3,7,8-tetrachlorodibenzo-p-dioxin [30]. Recent studies had shown that that CYP1B1, a newly identified member of the CYP1 family, plays a very important role in the metabolic activation of PAHs [31], [32]. Whether such cytochrome P450 dependent enzymes play also a role in tattooed skin is not investigated so far. The number of tattooed individuals substantially increased during the past 10 to 20 years. In light of the latency period of tumorigenic process, future epidemiological studies, which include a sufficient number of participants with and without tattoos, can help to answer the question whether skin malignancies are correlated to tattoos or not.

It has been suggested that ultraviolet radiation, a photoallergic effect, a persistent inflammatory reaction, or even trauma may promote malignant transformation [33]. This suggestion is supported by the fact that many of PAH such as benz[a]anthracene are not only mutagenic but also generate reactive oxygen species. When tattooed skin is exposed to solar radiation, the ultraviolet part of the spectrum can be absorbed in the present benz[a]anthracene, which generate singlet oxygen with a quantum yield of 85% [16], [19].

### Transportation via lymphatic system

After tattooing and wound healing, tattoo ink ingredients will stay in skin or will distribute in the human body, particularly the regional lymph nodes [34].Using an animal model, we previously determined a decrease of red tattoo pigments of about 30% six weeks after tattooing, which is assumed to be due to transportation processes in the body [19].Due to its hydrophobic nature, Carbon Black as fundamental matrix of the ready-to-use suspensions acts as strong sorptive phase for large conjugated aromatic systems such as PAH [35]. Nevertheless, PAH in tattoo inks can either be bound to Carbon Black or can be dissolved in the solvents of the inks suspension. Thus, PAH should appear in the lymph nodes via two processes. Firstly, unbound PAH in tattoo inks could be transported away from skin via lymphatic systemdirectly after tattooing. It is already known that concentration of tattoo inks in skin decrease over years after tattooing by up to 80% [36].Secondly, also the main substance in black tattoo inks - Carbon Black – is transported to the lymph nodes that can be easily seen by the deep black colour of all investigated lymph nodes.It is well known that PAH eagerly adsorb to Carbon Black surfaces and thereby an additional amount of PAH is carried to the lymph nodes. In the lymph nodes, these PAH molecules could be released from Carbon Black to an unknown extent and with an unknown time kinetic. It is assumed that the time kinetics might range from months to years [20].

As already discussed for the skin, epidemiological studies are necessary to decide whether transportation of unbound and adsorbed PAH via Carbon Black may pose a health risk on humans or not.

### Possible involvement of other organs

**Figure 4:**
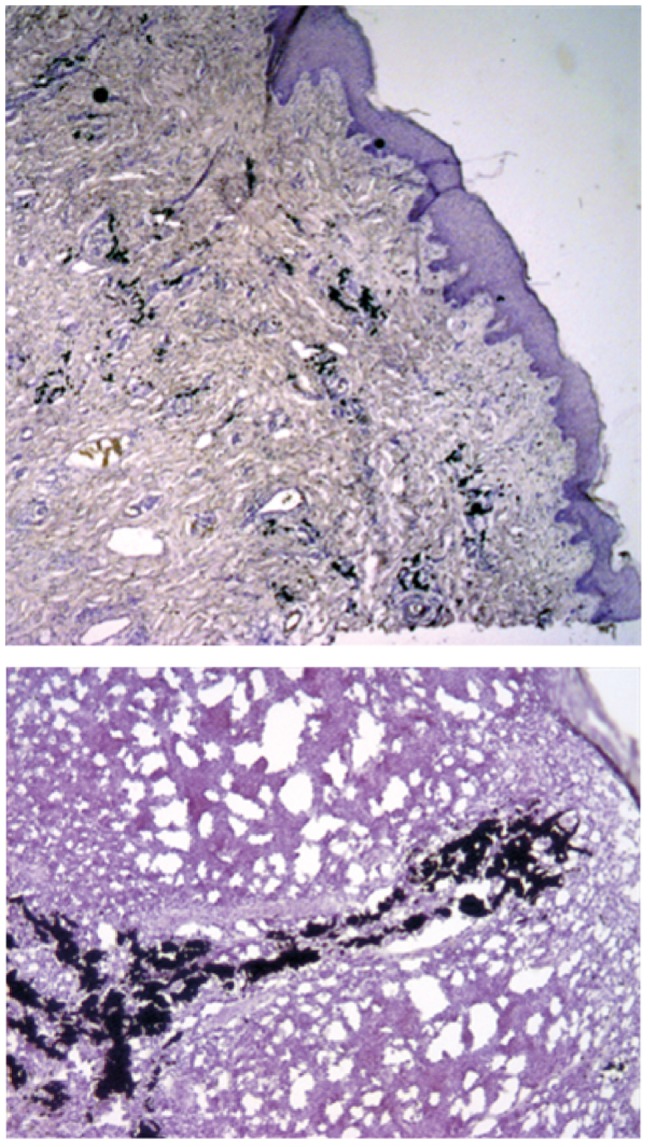
Histology of skin (top) and from the corresponding lymph node (bottom). The specimens were embedded in paraffin and, after hematoxylin-eosine (H&E) staining, imaged with a Zeiss Axiostar Plus microscope (10 fold optical magnification). The images show black tattoo particles, which are located inside the dermis or lymph node along with various PAH species as detected by extraction and chemical analysis together with adsorbed hazardous PAH.

Black ink particles can be easily found in skin and lymph nodes using histology and conventional light microscopy (Figure4). However, any further distribution of the black too ink particle in the human body after tattooing is unexplored so far. Beside the lymph nodes_ENREF_25, also other organs such as liver, spleen and kidney could be a destination of black ink particles depending of the route of transportation via lymphatic or blood vessel system. Thus, after tattooing the skin, the injected PAH may also pose a risk on other organs in the human body.

In light of the high concentrations of PAH in black tattoo inks [16], which are injected in the human body, the remaining concentrations in skin and lymph nodes are not surprising. However, these numbers also show that a large part of injected PAH should have been either metabolized in skin or transported away to other anatomical locations. This is of great importance because then PAH may cause deleterious effects elsewhere in the human body.

Recently, placental concentrations of PAH were analyzed by gas chromatography–mass spectrometry. PAH concentrations above the median as detected in some cases were associated with a 4.52-fold increased risk for any neural tube defects, and 5.84- and 3.71-fold increased risks for anencephaly and spina bifida, respectively [4].PAH is an important class of environmentally prevalent xenobiotics that can activate oxidative and electrophilic signaling pathways in lymphoid and non-lymphoid cells, including myeloid, epithelial, and other cells [37].Beside toxicity and mutagenicity [2], [22], PAH are also known to cause immunotoxic effects particularly IgE regulation that additionally involves the non-classified PAH such as phenanthrene [38]. Large quantities of phenanthrene were detected in skin and lymph nodes (Table 2).PAH reduce fertility, which may reflect altered survival of ovocytes [39], favour the development of cardiovascular diseases and trigger apoptosis in various cell lines [40]. However, PAH can also induce oxidative lesions resulting from the production of reactive oxygen species (ROS) [41].

## Conclusion

Millions of people worldwide have tattoos, which mainly consist of black inks contaminated with hazard substances such as PAH. In a recent survey in German speaking countries revealed that 28% of tattooed individuals have more than four tattoos and 36% tattoos have tattoos, which are larger than 900 cm^2^. Tattooed individuals, in particular with many and large tattoos, received up to several grams of black tattoo inks. Along with Carbon Black, by-products and impurities of the inks like PAH were placed in the skin. It is assumed that dissolved molecules and small Carbon Black particles of the injected ink left the skin via lymphatic or blood vessel systemdirectly after tattooing. Thus, these substances were distributed in the human body and the deep black lymph nodes are a clear evidence of that transportation process. The PAH concentrations found in the lymph nodes might be attributed to PAH molecules, which are adsorbed to the surface of Carbon Black particles. Such transportation processes were already studied in an animal model using PAH of diesel exhaust [20]. These PAH molecules in tattoo inks might cause health problems not only in skin but also in any other organs in the human body that is reached by these transportation processes.Thus, we highly recommend pharmacological, toxicological and epidemiological studies to clarify the possible impact of tattooing on human health.
